# Postoperative delirium in critically ill surgical patients: incidence, risk factors, and predictive scores

**DOI:** 10.1186/s12871-019-0694-x

**Published:** 2019-03-20

**Authors:** Onuma Chaiwat, Mellada Chanidnuan, Worapat Pancharoen, Kittiya Vijitmala, Praniti Danpornprasert, Puriwat Toadithep, Chayanan Thanakiattiwibun

**Affiliations:** 1 Department of Anesthesiology, Faculty of Medicine, Siriraj Hospital, Mahidol University, Bangkok, 10700 Thailand; 2 Division of Critical Care Medicine, Department of Medicine, Faculty of Medicine, Siriraj Hospital, Mahidol University, Bangkok, Thailand; 3 Integrated Perioperative Geriatric Excellent Research Center, Faculty of Medicine, Siriraj Hospital, Mahidol University, Bangkok, Thailand

**Keywords:** Postoperative delirium, Surgery, Intensive care unit (ICU), Risk factors

## Abstract

**Background:**

A common postoperative complication found among patients who are critically ill is delirium, which has a high mortality rate. A predictive model is needed to identify high-risk patients in order to apply strategies which will prevent and/or reduce adverse outcomes.

**Objectives:**

To identify the incidence of, and the risk factors for, postoperative delirium (POD) in surgical intensive care unit (SICU) patients, and to determine predictive scores for the development of POD.

**Methods:**

This study enrolled adults aged over 18 years who had undergone an operation within the preceding week and who had been admitted to a SICU for a period that was expected to be longer than 24 h. The CAM − ICU score was used to determine the occurrence of delirium.

**Results:**

Of the 250 patients enrolled, delirium was found in 61 (24.4%). The independent risk factors for delirium that were identified by a multivariate analysis comprised age, diabetes mellitus, severity of disease (SOFA score), perioperative use of benzodiazepine, and mechanical ventilation. A predictive score (age + (5 × SOFA) + (15 × Benzodiazepine use) + (20 × DM) + (20 × mechanical ventilation) + (20 × modified IQCODE > 3.42)) was created. The area under the receiver operating characteristic (ROC) curve (AUC) was 0.84 (95% CI: 0.786 to 0.897). The cut point of 125 demonstrated a sensitivity of 72.13% and a specificity of 80.95%, and the hospital mortality rate was significantly greater among the delirious than the non-delirious patients (25% vs. 6%, *p* < 0.01).

**Conclusions:**

POD was experienced postoperatively by a quarter of the surgical patients who were critically ill. A risk score utilizing 6 variables was able to predict which patients would develop POD. The identification of high-risk patients following SICU admission can provide a basis for intervention strategies to improve outcomes.

**Trial registration:**

Thai Clinical Trials Registry TCTR20181204006. Date registered on December 4, 2018. Retrospectively registered.

**Electronic supplementary material:**

The online version of this article (10.1186/s12871-019-0694-x) contains supplementary material, which is available to authorized users.

## Background

Delirium, a disturbance of consciousness which is both acute and fluctuating, is characterized by the lessened ability of an individual to focus, maintain, or shift attention; it is also associated with cognitive changes and disruptions in perception that are secondary to a general medical condition [1]. Delirium is an extremely common condition among hospitalized patients. Its incidence varies with the study population, but higher rates are observed among geriatric [2], post-surgical [3] and intensive care unit (ICUs) patients [4] Postoperative delirium (POD) among patients who have been treated with surgery and anesthesia is typically found during the first 3 postoperative days [1].

The incidence of POD was found to be 15%–25% among major elective surgery cases and up to 50% in procedures carrying an elevated risk, for example, cardiac surgery and fractures of the hip [5]. Although the duration of POD can be transient, it is linked to poor outcomes, such as a longer stay in post anesthesia care units (PACUs), ICUs, and hospitals; and higher medical complication rates and mortality levels [1, 6]. In the case of critically ill patients, delirium is a common adverse event, with a reported ICU incidence between 45 and 87% [7–9]. The incidence seems to be related to whether or not the population under study is comprised wholly of patients who are mechanically ventilated. Among non-intubated patients in an ICU, Van Rompaey et al. established a delirium incidence of only 20% [10], yet it reached 83% among those patients receiving mechanical ventilation [11].

In Thailand, data relating to postoperative delirium as well as delirium among critically ill patients are limited. In a study of the incidence of, and risk factors for, delirium among older patients who had been admitted for hip surgery at a single academic hospital, Muangpaisan et al. [12] found that delirium occurred equally during the pre- and postoperative periods (22.5%). Their study also determined that the risk factors for delirium were age, premorbid function, dementia/cognitive impairment, receiving NSAIDs around the clock, and postoperative sedative use. Moreover, the delirium incidence reported by Limpawattana et al. during a recent study at a medical ICU in northeastern Thailand [13] was 44% among older critically ill medical patients. The independent factors that they found related to the development of delirium were the use of physical restraints, a history of stroke, and multiple bed changes. As to the critically ill surgical population, a multicenter, prospective cohort study conducted at 9 academic institutions across Thailand reported a delirium incidence in surgical ICUs of only 3.6% (162/4450, 95% CI 3.09%–4.19%) [14]. Unfortunately, only a single assessment of delirium was performed each day (much less often than advised by guidelines) [15], and the correlation among the delirium assessors was not verified. These 2 factors could have resulted in the incidence being underestimated.

Given that information regarding delirium among surgical patients who are critically ill is very limited, research utilizing a better methodology to determine its rate of occurrence and the related risk factors in the surgical intensive care unit (SICU) population is needed. Thus, this research set out to establish the incidence, risk factors, and outcomes of postoperative delirium among critically ill SICU patients. In addition, the aim of this study was to develop a delirium prediction model that would enable the identification of high-risk patients in need of some form of intervention to lower the severity, and/or the adverse outcomes, of delirium.

## Materials and methods

### Design

A prospective, observational, cohort study was conducted. The Institutional Review Board of the Faculty of Medicine, Siriraj Hospital, Mahidol University, granted approval to conduct the research (IRB No. Si 718/2015).

### Study population

The study population included patients who were at least 18 years of age and were admitted to a SICU at Siriraj Hospital within 7 days of surgery [3], with that SICU stay anticipated to exceed 24 h. We excluded those SICU patients that had not undergone any operations; had communication problems (namely, being unable to communicate in Thai, or having a severe visual or auditory impairment that interfered with communication); or had a Richmond Agitation Sedation Scale (RASS) score of − 4 or − 5 during the whole of their period in the ICU. Data were collected between February 2016 and February 2017.

### Measurement instruments and data collection

Delirium was defined by the fulfillment of the CAM − ICU criteria (Thai version), namely, that a patient has: 1) acute onset and fluctuating symptoms, 2) inattention, and either 3) an altered level of consciousness or 4) disorganized thinking [16]. The Thai version of CAM − ICU has demonstrated satisfactory validity and reliability (specificity 94.7%, sensitivity 92.3%, and interrater reliability Cohen’s κ = 0.81) [17]. Moreover, it has shown practical feasibility when diagnosing delirium in a SICU setting.

The predisposing and precipitating factors potentially linked to the onset of delirium were grouped as preoperative, intraoperative, and postoperative variables. The preoperative risk factors included demographic variables obtained from a review of an individual patient’s medical records and interviews with any proxies. Each patient’s cognitive status was measured using the Modified Informant Questionnaire on Cognitive decline in the Elderly (modified IQCODE) [18].

Dementia was defined by a modified IQCODE score of more than 3.42 (sensitivity 90%, specificity 95%, positive predictive value (PPV) 0.94, and negative predictive value (NPV) 0.90) [18]. The intraoperative variables were obtained from anesthetic records, and they included the surgical site (abdomen, vascular, orthopedic, urological, gynecological, and head and neck), surgical type (emergency or elective), operation time, intraoperative blood loss, amount of blood transfused, and total fluid intake. Intraoperative hypotension was taken to be either a systolic pressure below 90 mmHg or the need to be treated with medications. Intraoperative hypoxemia was defined as oxygen saturation, derived from pulse oximetry, of below 90% for any duration.

The postoperative variables were primarily obtained from the SICU data records. They included the use of mechanical ventilation, physical restraints, or a Foley’s catheter; the presence of sleep deprivation or shock; exposure to psychoactive drugs (benzodiazepines, opioids and sedative); and the presence of coma (indicated by a RASS score of − 4 or − 5). The severity of illness at SICU admission was evaluated using the Acute Physiology and Chronic Health Evaluation II (APACHE II) and the Sequential Organ Failure Assessment (SOFA) scales. The delirium subtypes were recorded as hypoactive (RASS − 1 to − 3), hyperactive (RASS + 1 to + 4), and mixed type (hypo- and hyperactive) [19].

The postoperative outcomes comprised SICU adverse events (self-extubation; or the self-removal of a peripheral or central intravenous line, Foley’s catheter, or a nasogastric tube); episodes of nosocomial infection (with the new infectious episode occurring after 48 h of ICU admission, and being determined by either culture results or the clinical judgment of the attending physicians); the period of any mechanical ventilation; the duration of the stay in the ICU as well as the hospital; and the mortality rate within the hospital.

### Procedure

Delirium was evaluated at least twice daily (once during the 12 h from 6.00 am, and once in the 12 h after 6.00 pm), and whenever patients developed a mental change. Delirium was screened routinely utilizing a 2-step process. Initially, the patients’ level of consciousness was assessed by RASS; if the score was anywhere between − 3 and + 4, the evaluators progressed to the second step. However, in the event of a − 4 (responsive only to physical stimulus) or a − 5 (unresponsive to physical and verbal stimulus) RASS score in the first step, step 2 (the assessment of the patient with the Thai version of CAM − ICU) would not be performed. In addition, if a patient was found to be sedated in the first step, the dose of the sedative medication was adjusted and the patient was later re-assessed when a RASS score of − 3 or higher was achievable. The second step involved the determination of the patient’s delirium level using the Thai version of CAM − ICU, employing standard methodology. The assessments commenced on the first day after the patient’s operation and continued for 7 days, or until either discharge from the ICU or the death of the patient. Patients with delirium were further assessed until the CAM − ICU was negative for 24 h. Thereafter, the ICU attending physician was notified for further management.

The trained clinical researchers were the physicians OC, PD, and PT, who had been trained by a nurse who had been the principal investigator (Sirirat Mueankwan) of the Thai CAM − ICU validation study [17]. Thereafter, the 3 physicians trained the SICU nurses who had 5 or more years’ nursing care experience. To ensure reliability among the assessors, inter-rater reliability scores were calculated. When the kappa score reached 0.8, the trained nurses could then perform the Thai CAM − ICU assessments.

### Statistical analyses

The sample size was estimated based on multiple logistic regression analysis [20]. Based on literature review, the risk factors during intraoperative and postoperative period were approximately 10 variables [21, 22] and the number of patients with delirium should be 5 to 10 times of risk factors. Therefore, 100 patients with delirium were required. With the reported incidence of POD about 44% [3], 227 patients were needed. To compensate for 10% dropout, sample size was increased for 250 cases.

The clinical reliability of the delirium assessments was analyzed using the kappa coefficient statistics. Every SICU nurse received training to get kappa > 0.8 before commencing data collection. Demographic variables were presented as mean ± standard deviation and median (interquartile range) for continuous data, and frequency and percentage for categorical data. Group comparisons were performed by using the independent Student t-test, Mann–Whitney U test, chi-square test, or Fisher’s exact test, as appropriate. A prediction model was developed by using logistic regression and by evaluating the degree of association of each potential prognostic determinant with the presence or absence of delirium. Eleven risk factors with univariable *P*-value less than 0.2 including age, dementia defined by a modified IQ-code ≥3.42, previous stoke, diabetes mellitus (DM), hypertension, serum creatinine, SOFA score, active infection, emergency surgery, benzodiazepine use, and the use of mechanical ventilation were entered into multiple logistic regression. With those factors, a multivariate logistic regression analysis with enter elimination was utilized to appraise the independent variables associated with delirium development. A predictive model was then developed based on regression coefficients from the final multivariate model. The Hosmer–Lemeshow statistic was used to assess the model’s calibration, or its fit to the data; this was based on the degree of agreement between the predicted risk-score probabilities using the model and the actual observed probabilities.

The model’s prognostic ability to discriminate between individuals who had, and did not have, delirium was estimated by using the receiver operating characteristic curve **(**ROC**)**. The estimated shrinkage factor for the performance of delirium was estimated. For internal validation study, we calculated a model based on a new ROC by using the internal bootstrap validation c-statistic to adjust for over fitting.

Finally, the ROC curve was presented to demonstrate the performance of delirium for the best cut-off point in terms of Youden’s index, sensitivity, specificity, PPV, NPV, positive likelihood ratio **(**LR+**)**, negative likelihood ratio **(**LR-**)**, area under curve **(**AUC**)**, and 95**%** confidence interval. The Youden’s index was the difference between the true and the false positive rates. Maximizing this index allows an optimal cut-off point to be found, from the ROC curve, independently from the prevalence [23, 24]. Statistics were analyzed using SPSS Statistics for Windows, version 18 (SPSS Inc., Chicago, IL, USA); R statistic, version 3.4.0; and MedCalc Statistic Software, version 17.6 (MedCalc Software BVBA, Ostend, Belgium).

## Results

**Fig. 1:**
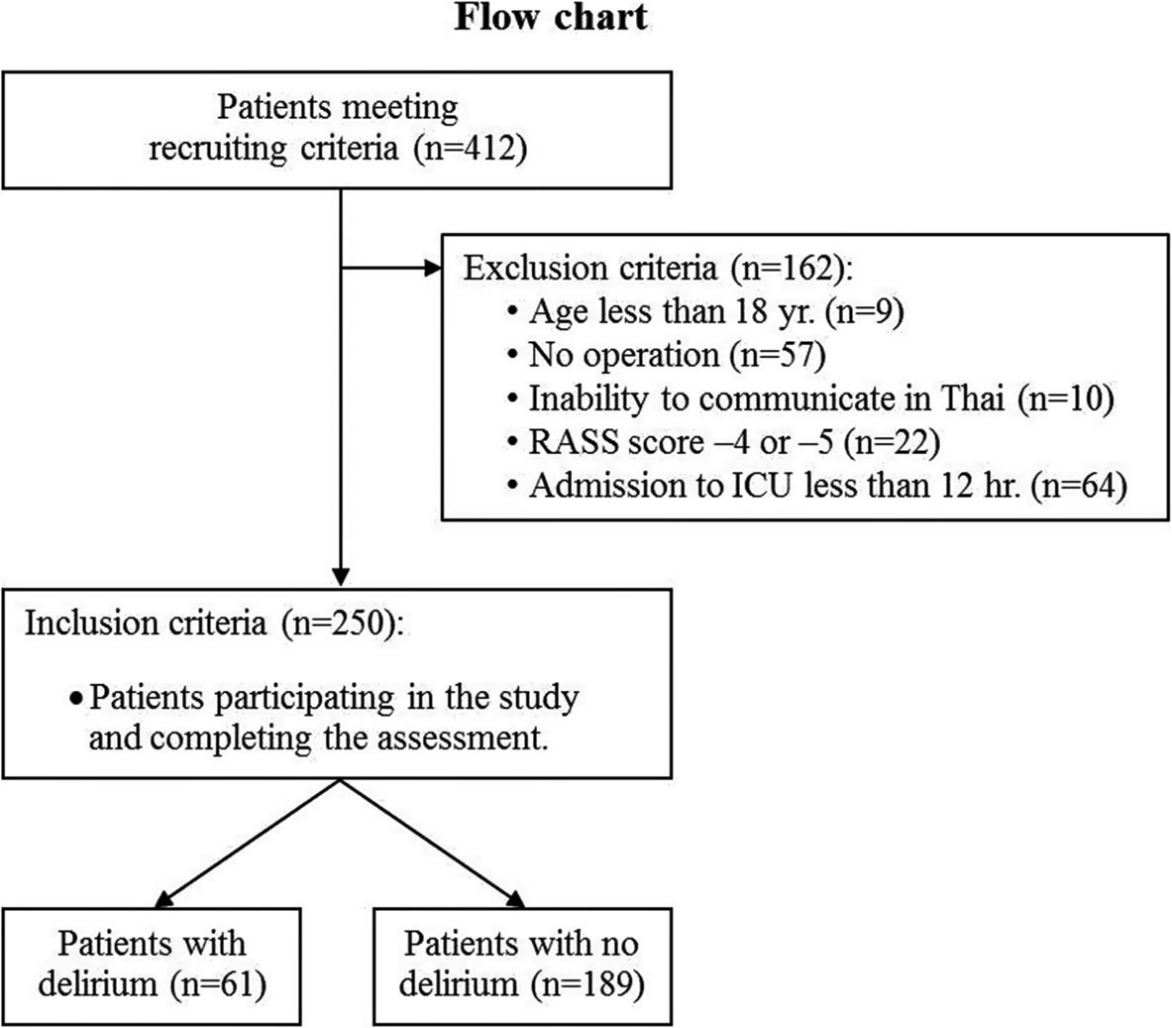
Flow chart of patient enrollment

**Fig. 2:**
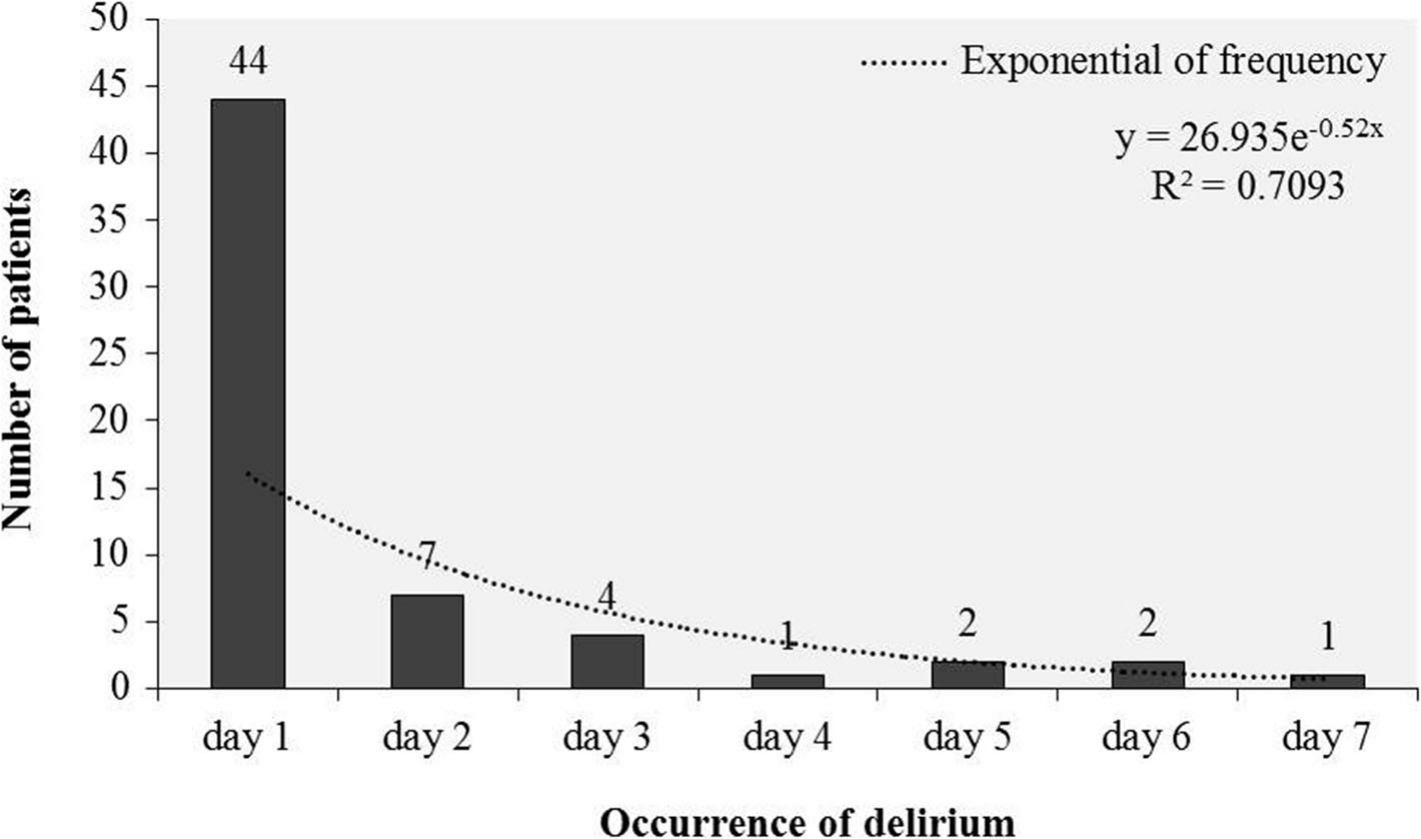
Occurrence of delirium (day after SICU admission)

Of the 412 recruited patients, a total of 162 were excluded for the reasons showed in Fig. 1. As a result, 250 patients were enrolled, 61 of whom (24.4%) developed delirium according to the CAM − ICU assessment (Fig. 1). After their SICU admission, the majority of patients (72%) were found to have delirium on day 1 (Fig. 2). The hypoactive subtype was found in 44/61 patients (72%), followed by the mixed and hyperactive subtypes of delirium, representing 15 and 13% respectively.

### Postoperative risk factors

The patients’ baseline characteristics are at Table 1. The delirious patients were markedly older than the non-delirious patients, with 90% of those with delirium being older than 60 years. The proportions of patients with DM, hypertension, previous stroke, and dementia **(**as assessed by the modified IQ code**)** were significantly larger among the delirious than the non-delirious patients. Moreover, the delirious patients had undergone a significantly greater number of emergency procedures than had the non-delirious patients. However, during surgery, the delirious and non-delirious patients did not demonstrate any significant differences in the types of surgery, operating times, blood loss levels, red cell transfusions, or intraoperative events **(**including hypoxia and hypotension**)**. With regard to the SICU admission data, the patients who developed delirium demonstrated a significantly higher severity, as assessed by the APACHE II and SOFA scores; higher levels of serum BUN, creatinine, and serum sodium; and a higher number of sepsis cases on SICU admission. As to medication use, higher amounts of preoperative and postoperative benzodiazepine were administered to the delirious patients. The patients who had delirium were also more likely to be administered propofol during their SICU stay than were those without delirium.

**Table 1:** Baseline characteristics of delirious and non-delirious patients

Variables	Delirium (*n* = 61)	No delirium (*n* = 189)	*P*-value
Demographic data			
Age (years)	72.7 ± 11.4	61.4 ± 16.8	< 0.001
≥ 60	55 (90.2%)	114 (60.3%)	< 0.001
Gender			
Male	31 (50.8%)	90 (47.6%)	0.768
Comorbidities			
Hypertension	50 (81.9%)	105 (55.6%)	< 0.001
DM	26 (42.6%)	37 (19.6%)	0.001
Cardiac disease	21 (34.4%)	43 (22.8%)	0.091
Previous stroke	15 (24.6%)	18 (9.5%)	0.004
Modified IQCODE score ≥ 3.42	10 (16.39%)	6 (3.2%)	0.001
ESRD or CKD stage 4–5	10 (16.4%)	24 (12.7%)	0.520
Cirrhosis	3 (4.9%)	9 (4.8%)	1.000
Smoking history pack year	41.9 ± 27.1	24.4 ± 21.6	0.155
≥ 30 pack year	10 (16.4%)	20 (10.6%)	0.259
Current alcohol consumption	6 (9.8%)	11 (5.8%)	0.378
Coma	17 (27.9%)	16 (8.5%)	< 0.001
Intraoperative data			
Emergency Surgery	34 (55.74%)	74 (39.2%)	0.026
Vascular surgery	20 (32.8%)	32 (16.9%)	0.011
Non-vascular surgery			
Intra-abdominal	23 (37.7%)	65 (34.4%)	0.646
Orthopedic	3 (4.9%)	26 (13.8%)	0.068
Gynecological	3 (4.9%)	23 (12.2%)	0.147
Other	12 (19.7%)	43 (22.8%)	0.723
Operation time	193.5 ± 162.6	234.8 ± 178.9	0.111
Intraoperative blood loss (mL)	250 (60–700)	400 (100–1400)	0.079
Intraoperative PRC transfusion (mL)	264 (0–663)	0 (0–1023)	0.865
Hypoxia	3 (4.9%)	7 (3.7%)	0.710
Intraoperative hypotension	50 (82.0%)	146 (77.3%)	0.480

*ESRD*, end stage renal disease; *CKD*, chronic kidney disease; *DM*, diabetes mellitus; *Modified IQCODE score*, modified informant questionnaire on cognitive decline in the elderly score

Data presented as mean ± SD or median (IQR) or N (%)

Eleven significant and relevant factors were entered into the multivariate analysis. The factors that remained independently associated with delirium were age, dementia, as defined by an average score of the modified IQ CODE ≥3.42, DM; SOFA scores, preoperative and postoperative benzodiazepine use, and the use of mechanical ventilation in the SICU. AOR and 95% CI were showed in Table 2. Dementia, defined by a modified IQ code ≥3.42, remained in the model even though the *P*-value > 0.05 because most previous studies [3, 4, 25] demonstrated that dementia was strongly related to the occurrence of delirium. In addition, the AUC of dementia defined by a modified IQ code ≥3.42 was 0.57, which was not very different from the other significant variables.

**Table 2:** Variables of prediction model and regression coefficients

Variables	β	Adjusted odds ratio (95% CI)	*P*-value
Age (years)	0.057	1.06 (1.03–1.09)	< 0.001
SOFA score	0.230	1.26 (1.12–1.42)	< 0.001
Benzodiazepine use (perioperative)	0.813	2.26 (1.08–4.69)	0.029
DM	1.109	3.03 (1.43–6.44)	0.004
Mechanical ventilation	1.178	3.25 (1.19–8.87)	0.022
Modified IQCODE score ≥ 3.42	1.219	3.38 (0.94–12.12)	0.061

*Modified IQCODE score*, modified informant questionnaire on cognitive decline in the elderly score; *DM*, diabetes mellitus; *SOFA score*, sequential organ failure assessment score

### Development of the prediction model for postoperative delirium

A prediction model was derived from a multiple logistic regression using significant risk factors from the final analysis (Table 2). The final formula requires 6 factors (2 quantitative factors and 4 binary factors). All regression coefficients in the final equation were divided by the lowest coefficient. The coefficient of each factor was 4, 14, 19 and 21, respectively. Subsequently, a binary logistic regression models were fitted:

$$ \mathrm{Age}+\left[\begin{array}{c}\left(4\times \mathrm{SOFA}\ \mathrm{score}\right)+\left(14\times \mathrm{Benzodiaze}\ \mathrm{pine}\ \mathrm{use}\right)+\left(19\times \mathrm{DM}\right)+\\ {}\left(21\times \mathrm{Mechanical}\ \mathrm{ventilato}\ \mathrm{r}\right)+\left(21\times \mathrm{Modiflied}\ \mathrm{IQCODE}\ \mathrm{score}\ge 3.42\right)\end{array}\right] $$

The simplified coefficient of each factor was rounded to integer like 5, 15 and 20 to ease the calculation. The formula of the prediction model was:

$$ \mathrm{Age}+\left[\begin{array}{c}\left(5\times \mathrm{SOFA}\ \mathrm{score}\right)+\left(15\times \mathrm{Benzodiaze}\ \mathrm{pine}\ \mathrm{use}\right)+\left(20\times \mathrm{DM}\right)+\\ {}\left(20\times \mathrm{Mechanical}\ \mathrm{ventilato}\ \mathrm{r}\right)+\left(20\times \mathrm{Modiflied}\ \mathrm{IQCODE}\ \mathrm{score}\ge 3.42\right)\end{array}\right] $$

**Fig. 3:**
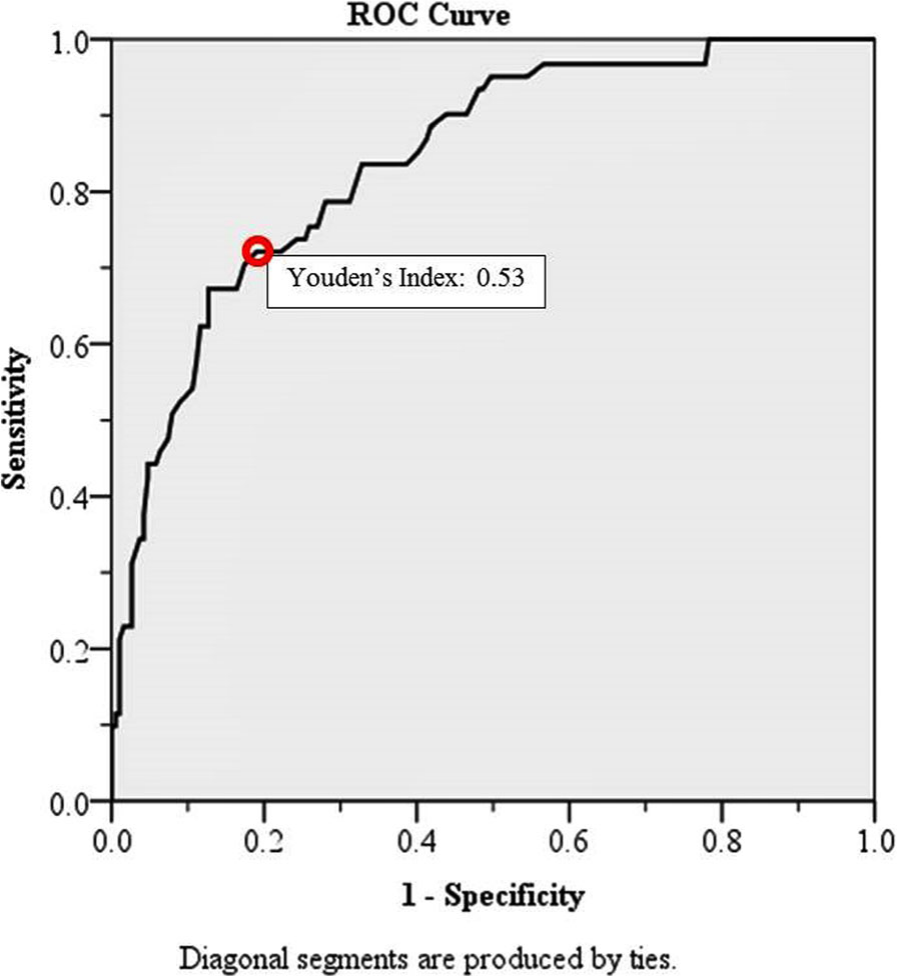
Delirium predictive score ROC curve

The simplified equation was selected because of the clinically meaningful of the factors with an assessed fit to the data fitted by the Hosmer-Lemeshow goodness-of-fit test of 0.389, which *P*-value > 0.05 and a − 2 Log-Likelihood of 200.682 was shown that the model fits the data well. In addition, the ROC of the prediction model was generated using predicted probabilities from the logistic regression model; the AUC of simplified equation (AUC 0.84, 95% CI: 0.786–0.897) was not different from the first equation (AUC 0.84, 95% CI: 0.788–0.899). Furthermore, the internal bootstrap validation c-statistic of the same population was evaluated with the value of 0.82 **(**over fitting = 0.2**)**. The accuracy of the model was then tested with the estimated shrinkage factor, which demonstrated as 0.92, as shown at Fig. 3.

The optimum cut-off point to discriminate between a high and low probability of postoperative delirium was 125. This point presented the highest value of Youden’s index (0.53), the best AUC, and the optimum value of sensitivity (72.13%) and specificity (80.95%), with a positive predictive value (PPV) of 55.0 and a negative predictive value (NPV) of 90.0 (Table 3). The demonstration of the calculation of predictive model was described in the appendix (Additional files 1 and 2).

**Table 3:** Receiver operating characteristic curve analysis

Cut point	Sensitivity (%)	Specificity (%)	PPV	NPV	LR+	LR-	AUC (95% CI)	Youden’s Index
≥ 110	83.61	62.96	42.15	92.25	2.26	0.26	0.73 (0.66–0.80)	0.47
≥ 115	78.69	70.37	46.15	91.10	2.66	0.30	0.75 (0.68–0.82)	0.49
≥ 119	75.41	73.02	47.42	90.20	2.79	0.34	0.74 (0.67–0.82)	0.48
**≥ 125**	**72.13**	**80.95**	**55.00**	**90.00**	**3.79**	**0.34**	**0.77 (0.69–0.84)**	**0.53**
≥ 129	67.21	86.24	61.19	89.07	4.89	0.38	0.77 (0.69–0.84)	0.53
≥ 133	57.38	88.89	62.50	86.60	5.16	0.48	0.73 (0.65–0.81)	0.46

*PPV*, positive predictive value; *NPV*, negative predictive value; *LR+*, positive likelihood ratio; *LR-*, negative likelihood ratio; *AUC*, area under curve; *95% CI*, 95% confidence interval

The optimum cut-off point to discriminate between a high and low probability of postoperative delirium was 125 with the highest Youden'd Index, the best AUC and the optimum sensitivity and specificity

### Adverse events

Delirious patients demonstrated significantly longer periods of mechanical ventilation, as well as longer ICU and hospital lengths of stay. Higher numbers of ICU events, including self-extubation and self-removal of catheters **(**intravenous, Foley’s, and nasogastric tubes**)**, were found among the patients who developed POD, and they had higher percentages for the use of physical restraints and the incidence of sleep deprivation. In addition, delirious patients were more likely to develop pneumonia and surgical site infections. Finally, the ICU and hospital mortality rates were higher among the patients who had delirium (Table 4).

**Table 4:** Outcomes

Variables	Delirium (*n* = 61)	No delirium (*n* = 189)	*P*-value
Duration of mechanical ventilation (days)	3 (1–6)	1 (0–3)	< 0.001
ICU events			< 0.001
None	45 (73.8%)	181 (95.8%)	
Self-extubation	8 (13.1%)	3 (1.6%)	
Self-removal of Foley’s catheter/NG tube	8 (13.1%)	5 (2.7%)	
Physical restraint	35 (57.3%)	26 (13.8%)	< 0.001
Sleep deprivation	18 (29.5%)	12 (6.4%)	< 0.001
Nosocomial infection in ICU	11 (9.5%)	18 (18.0%)	0.105
ICU length of stay (days)	5 (3–8)	2 (1–4)	< 0.001
ICU mortality	7 (11.5%)	2 (1.1%)	0.001
Hospital length of stay (days)	19 (13–33)	15 (10–25)	0.009
Hospital mortality	15 (24.6%)	11 (5.8%)	< 0.001

*ICU*, intensive care unit; *UTI*, urinary tract infection; *CRBSI*, catheter-related bloodstream infection

Data presented as mean ± SD or median (IQR) or N (%)

## Discussion

The cohort of critically ill surgical patients in this study displayed an incidence of POD of 24.4**%**. The independent risk factors associated with POD were a higher age, dementia, underlying DM, higher severity scores at time of the SICU admission, the use of benzodiazepine medication during the perioperative period, and the use of mechanical ventilation during the ICU stay. A predictive score to identify patients who had a high potential to develop delirium was created. An internal validation which was then performed in the same population demonstrated high accuracy. The cut-off point of 125 showed high sensitivity and specificity. In addition, the delirious patients demonstrated worse clinical outcomes than the non-delirious patients.

The reported incidences of POD among surgical patients range from very low to high percentage [25]. The variations in those rates have been related to the studied population, the surgical procedures, and the delirium assessment tools employed [26, 27]. Although POD is common in older surgical patients, it can occur among patients of all ages if the recognized precipitating risk factors are present, for example, major [28] or emergency surgery [11, 29]. In the present study, delirium occurred in a quarter of the study population; however, 90**%** of the POD cases were found among those patients aged over 60. In addition, those patients who required emergency or major surgery **(**including vascular and orthopedic surgery**)** had a higher incidence of POD than patients undergoing elective or other types of surgery.

Delirium can present as hyperactive, hypoactive, or as mixed forms, and RASS is used to categorize the subtype of delirium, as previously mentioned [30]. The majority of delirious patients (72%) in this cohort were hypoactive. Previous studies [30, 31] have demonstrated that increased age is associated with hypoactive delirium; the prognosis seems to be worse with this type because of an under- recognition by healthcare personnel, resulting in delayed treatment. In addition, differentiating delirium from dementia or depression can be confusing; as well, a patient might even have all of these psychiatric syndromes at the same time [32]. Given a lack of information regarding the history of a patients’ baseline mental status both in medical records and from family members, it is safest to assume delirium [33]. A combination of non-pharmacological and pharmacological interventions to manage and prevent further complications should be implemented. This includes behavioral and non-pharmacological strategies such as: making sure the patient has the required sensory enhancement devices (hearing aids, ensuring glasses), early mobilization, cognitive orientation, pain control, sleep enhancement and regular medication review [5]. When it comes to a pharmacologic approach for delirium prevention, the benefits of approach remain unclear and show no significant effect on length of hospital stay or mortality [34].

The POD risk factors for surgical patients who are critically ill have been addressed by several studies [35, 36]. The factors can be differentiated into 2 broad types: those linked to the patients (the predisposing factors), and those that induce the occurrence of delirium (the precipitating factors). Consequently, the overall risk for delirium results from a combination of the predisposing and precipitating factors. Older age, dementia, depression, multiple or specific comorbidities, and alcohol abuse have been demonstrated to be common predisposing risk factors [37]. Among the chronic diseases, cardiovascular [38, 39] and metabolic diseases, such as DM [40, 41], have been reported as being associated with POD the most often. In the current study, older age, dementia (assessed by the modified IQ code), and DM were the predisposing factors that were found to be independently associated with the development of delirium. Dementia was assessed both by the patient’s history and the assessment tool. The prevalence of dementia among the elderly delirious patients was 5 times higher when evaluated by the tool than when using information obtained from history taking. As dementia is an important risk factor, it is far more preferable to evaluate this condition using a validated and reliable tool rather than gathering data only via history taking. Among the precipitating factors, drugs (including psychoactive agents and sedative hypnotics), surgery, anesthesia, the severity of illness, infections, and the use of a mechanical ventilator were the most common [10]. In contrast to a previous report [42], the intraoperative risk factors (site of surgery, duration of surgery, bleeding, and hypoxia and hypotension during anesthesia) were not linked to POD development in the present study’s cohort. Differences in the types of the populations and the surgery types might be the reasons. All patients in the current study were admitted to a SICU post-operatively and nearly the majority had undergone intra-abdominal surgery, with an average duration of surgery of less than 4 h and minimal blood loss (< 500 ml). Not surprisingly, mechanical ventilation, perioperative benzodiazepine use, and illness severity were precipitating factors of delirium found among patients in the current study. A direct causal link between these factors and the occurrence of POD can, however, not be proven by this study.

As previously mentioned, both POD and delirium in general contributes to unfavorable clinical and functional outcomes. Strong evidence has indicated that POD is connected with higher mortality in both the short and long terms. POD’s impact on mortality does not depend on surgical type for either elective or emergency surgery. In the hospital, delirium increases the risks of adverse events and results in longer lengths of stay [37]. The current study reported an increased incidence of death, a prolonged length of stay, and a higher rate of adverse events among the delirious patients. ICU adverse events, including self extubation and the self-removal of catheters, can result in high morbidity, such as aspiration, infection and bleeding. The use of physical restraints and sleep deprivation can be either precipitating factors or the consequences of delirium. Nevertheless, in the present study, those two conditions were categorized as adverse outcomes of delirium (i.e., were considered as consequences) because the majority of the cohort experienced delirium very early (day 1 after their SICU admission). Delirium is also related to poor long-term outcomes; a meta-analysis [2] of 3000 patients who were followed for almost 2 years demonstrated that delirium that occurred in hospital was connected with an incident of dementia, a higher risk of death, and long term cognitive dysfunction even among patients who were aged under 50 [43].

Delirium can contribute to higher morbidity and mortality and a number of risk factors have been recognized in different populations. It would be much better if a simple and accurate delirium prediction score could be developed that can identify those critically ill surgical patients who have a high likelihood of developing POD, drawing on the known predisposing and the immediate precipitating factors. Although several scoring systems for predicting POD have been developed and used, some limitations exist in terms of their general application to critically ill surgical patients. For example, some of the prediction scores were developed for medical patients [44] or only for general surgical patients [45], and their prediction scores were too complicated [46]. As consequence, the prediction score for the critically ill, general surgical patients was developed using the information provided by the present study. Separating the delirious patients with an ROC curve showed an AUC of 0.84 and an estimated shrinkage factor of 0.92. Both values were classified as good [47, 48]. Therefore, the equation was suitable for discriminating between delirious and non-delirious patients. The ROC curve demonstrated an optimal cut-off point of 125, the point with the highest Youden’s index, with a sensitivity of 72.13% and a specificity of 80.95% [23]. Given that, we can demonstrate a simple predictive score with a high accuracy and reliability.

This study has several limitations. Firstly, the studied population included only surgical patients who had undergone general surgery and had been admitted to a SICU; the results therefore cannot be generalized to all critically ill surgical patients, for example, critically ill cardiac and neuro-surgical patients. In addition, some relevant information during the preoperative and intraoperative periods was not obtained, such as any history of previous incidents of delirium, intraoperative medication usage, the use of antipsychotic medications, and the degree of postoperative pain. Moreover, due to resource limitations, the frequency of delirium assessment could only be regularly performed twice a day. This might result in an underestimation of the incidence of delirium. Lastly, the management of delirium was not protocolized; the resulting variety in management might affect the outcomes. To illustrate, a consistent administration of some medications (such as dexmedetomidine) or of non-pharmacological interventions for some patients might have improved the outcomes. However, the management of hyperactive delirium by the SICU staff did not appear to differ much in practice, and most hyperactive delirious patients in the SICU were consulted by a geriatrician or psychiatrist.

## Conclusions

POD affected around a quarter of the study population in this prospective cohort study. Contributing peri-operative factors include: older patients, preoperative dementia, underlying DM, a high severity at SICU admission, the use of benzodiazepine, and the use of mechanical ventilation were determined to be related to the occurrence of postoperative delirium. Patients with diagnosed POD were at risk of increased mortality rates, prolonged hospital stays, and a higher number of SICU complications. A simple and validated predictive score was developed to identify those patients who had a high probability of developing postoperative delirium, and an external validation will be performed. Based on the current information, a delirium management protocol that encompasses prevention, detection, and treatment is required to improve patients’ care and to ameliorate the adverse clinical and functional outcomes.

## Additional files

Additional file 1: Appendix for delirium prediction. (DOCX 17 kb)

Additional file 2: **Figure S4.** Distribution of the predicted probability of delirious patients. (JPG 78 kb)

## Abbreviations

AOR: Adjusted odds ratio
APACHE II: Acute Physiology and Chronic Health Evaluation II
AUC: Area under curve
BUN: Blood Urea Nitrogen
CAM − ICU: The Confusion Assessment Method for the Intensive Care Unit
CKD: Chronic kidney disease
Cr: Creatinine
CRBSI: Catheter-related bloodstream infection
DM: Diabetes mellitus
ESRD: End stage renal disease
LR +: Positive likelihood ratio
LR-: Negative likelihood ratio
Modified IQCODE: Modified Informant Questionnaire on Cognitive decline in the Elderly
NPV: Negative predictive value
PACUs: Post Anesthesia Care Units
POD: Postoperative delirium
PPV: Positive predictive value
RASS: Richmond Agitation Sedation Scale
ROC: Receiver operating characteristic curve
SICU: Surgical Intensive Care Unit
SOFA: Sequential Organ Failure Assessment scales
UTI: Urinary tract infection
