# Correction to: Postoperative delirium in critically ill surgical patients: incidence, risk factors, and predictive scores

**DOI:** 10.1186/s12871-019-0732-8

**Published:** 2019-04-22

**Authors:** Onuma Chaiwat, Mellada Chanidnuan, Worapat Pancharoen, Kittiya Vijitmala, Praniti Danpornprasert, Puriwat Toadithep, Chayanan Thanakiattiwibun

**Affiliations:** 1 Department of Anesthesiology, Faculty of Medicine, Siriraj Hospital, Mahidol University, Bangkok, 10700 Thailand; 2 Division of Critical Care Medicine, Department of Medicine, Faculty of Medicine, Siriraj Hospital, Mahidol University, Bangkok, Thailand; 3 Integrated Perioperative Geriatric Excellent Research Center, Faculty of Medicine, Siriraj Hospital, Mahidol University, Bangkok, Thailand

**Correction to: BMC Anesthesiol (2019) 19:39**

**https://doi.org/10.1186/s12871-019-0694-x**

Following publication of the original article [1], the authors reported a missing data on Table 1 in their paper. The original article [1] has been updated.

The correct Table 1 is shown below.

**Table 1:** Baseline characteristics of delirious and non-delirious patients

Variables	Delirium (*n* = 61)	No delirium (*n* = 189)	*P*-value
Demographic data			
Age (years)	72.7 ± 11.4	61.4 ± 16.8	< 0.001
≥ 60	55 (90.2%)	114 (60.3%)	< 0.001
Gender			
Male	31 (50.8%)	90 (47.6%)	0.768
Comorbidities			
Hypertension	50 (81.9%)	105 (55.6%)	< 0.001
DM	26 (42.6%)	37 (19.6%)	0.001
Cardiac disease	21 (34.4%)	43 (22.8%)	0.091
Previous stroke	15 (24.6%)	18 (9.5%)	0.004
Modified IQCODE score ≥ 3.42	10 (16.39%)	6 (3.2%)	0.001
ESRD or CKD stage 4–5	10 (16.4%)	24 (12.7%)	0.520
Cirrhosis	3 (4.9%)	9 (4.8%)	1.000
Smoking history pack year	41.9 ± 27.1	24.4 ± 21.6	0.155
≥ 30 pack year	10 (16.4%)	20 (10.6%)	0.259
Current alcohol consumption	6 (9.8%)	11 (5.8%)	0.378
Coma	17 (27.9%)	16 (8.5%)	< 0.001
Intraoperative data			
Emergency Surgery	34 (55.74%)	74 (39.2%)	0.026
Vascular surgery	20 (32.8%)	32 (16.9%)	0.011
Non-vascular surgery			
Intra-abdominal	23 (37.7%)	65 (34.4%)	0.646
Orthopedic	3 (4.9%)	26 (13.8%)	0.068
Gynecological	3 (4.9%)	23 (12.2%)	0.147
Other	12 (19.7%)	43 (22.8%)	0.723
Operation time	193.5 ± 162.6	234.8 ± 178.9	0.111
Intraoperative blood loss (mL)	250 (60–700)	400 (100–1400)	0.079
Intraoperative PRC transfusion (mL)	264 (0–663)	0 (0–1023)	0.865
Hypoxia	3 (4.9%)	7 (3.7%)	0.710
Intraoperative hypotension	50 (82.0%)	146 (77.3%)	0.480
ICU admission			
Hemoglobin (mg/dL)	10.5 ± 2.5	10.9 ± 2.1	0.156
Blood sugar at admission (mg/dL)	155.6 ± 42.1	152.2 ± 46.3	0.611
Serum albumin (mg/dL)	2.7 ± 0.6	2.8 ± 0.7	0.168
BUN/Cr ratio > 20	22 (36.1%)	48 (25.4%)	0.139
Sepsis	24 (39.3%)	37 (19.6%)	0.003
APACHE II score	12.1 ± 4.8	8.3 ± 3.9	< 0.001
SOFA score	5.8 ± 3.4	3.4 ± 2.7	< 0.001
Mechanical ventilation	54 (88.5%)	131 (69.2%)	0.002
Medication			
Preoperative-Benzodiazepine	8 (13.1%)	10 (5.3%)	0.049
Inraoperative-Benzodiazepine	32 (53.3%)	93 (47.7%)	0.658
Benzodiazepine (Pre and Postoperative)	27 (44.3%)	36 (19.1%)	< 0.001
Preoperative-Opioid	5 (8.2%)	18 (9.5%)	1.000
Intraoperative-Opioid	58 (96.7%)	176 (94.1%)	0.740
Opioid (Pre and Postoperative)	59 (96.7%)	184 (97.4%)	1.000
Postoperative-Propofol use	20 (32.8)	31 (16.4%)	0.010

*ESRD* End stage renal disease, *CKD* Chronic kidney disease, *DM* Diabetes mellitus, *Modified IQCODE score* Modified informant questionnaire on cognitive decline in the elderly score, *BUN* Blood urea nitrogen, *Cr* Creatinine, *APACHE II score* Acute physiology and chronic health evaluation II score, *SOFA score* Sequential organ failure assessment score

Data presented as mean ± SD or median (IQR) or N (%)
