# Supplementary material for: Postoperative delirium in critically ill surgical patients: incidence, risk factors, and predictive scores
Source: BMC Anesthesiol. 2019 Mar 20;19:39. doi: 10.1186/s12871-019-0694-x (PMC6425578; doi:10.1186/s12871-019-0694-x)
Supplement: Supplementary file 1 — Appendix for delirium prediction. (DOCX 17 kb) [file 12871_2019_694_MOESM1_ESM.docx]

**Appendix**

To demonstrate the manner in which the prediction model of postoperative delirium described in this paper is used, we present two illustrative patients assessed with the following equation:

$$\text{Age+}\left[\begin{matrix} \text{(5}\times\text{SOFA score) + (15}\times\text{Benzodiazepine use) + (20}\times\text{DM) + } \\ \text{(20}\times\text{Mechanical ventilator) + (20}\times\text{Modiflied IQCODE score }\geq\text{ 3.42)} \end{matrix}\right]$$

Where Modified IQCODE Score (score 0 if Modified IQCODE < 3.42; score 1 if Modified IQCODE ≥ 3.42); DM, Mechanical Ventilator and Benzodiazepine Use (score 0 if No; score 1 if Yes). The optimal cut-off point was 125; patients who had scores beyond this point were associated with the development of postoperative delirium.

**Case 1**

A 79-year-old female patient with the diagnosis of gangrenous gall bladder was scheduled for percutaneous cholecystectomy. Her comorbidities included hypertension, stroke, DM, and coronary artery disease; the modified IQ code score was 3.13. The admission APACHE II and SOFA scores were 19 and 11, respectively. She was on mechanical ventilation and retained a Foley’s catheter. Midazolam and fentanyl were used for sedation and pain management.

The values for this patient were entered in the prediction model:

$$\begin{matrix} \text{Y}=79+\text{[}\left(5\times11\right)+\left(15\times1\right)+\left(20\times1\right)+\left(20\times1\right)+\left(20\times0\right)] \\ \text{Y}=\text{ 189} \end{matrix}$$

As the values of the discriminant function (Y) are over 125, the model correctly predicted delirium. Nevertheless, according to the predicted probability of the model, there was a 92.9% likelihood that this would occur (Figure 4).

**Case 2**

A 65-year-old female patient presented with necrotizing fasciitis at the right foot. She developed septic shock and was brought to the operating room for debridement. She had no comorbidities. The modified IQ code score was 3.06, the APACHE II score was 20, the SOFA score was 7, and she was on mechanical ventilation. Only fentanyl was used for sedation.

The values for this patient were entered in the prediction model:

$$\begin{matrix} \text{Y}=65+\text{[}\left(5\times7\right)+\left(15\times0\right)+\left(20\times0\right)+\left(20\times1\right)+\left(20\times0\right)] \\ \text{Y}=\text{ 120} \end{matrix}$$

As the values of the discriminant function (Y) are below 125, the model correctly predicted no delirium. According to the predicted probability of the model, there was only a 25.6% chance that delirium would occur (Figure 4).
