# Supplementary figures and images for: Postoperative delirium in critically ill surgical patients: incidence, risk factors, and predictive scores
Source: BMC Anesthesiol. 2019 Mar 20;19:39. doi: 10.1186/s12871-019-0694-x (PMC6425578; doi:10.1186/s12871-019-0694-x)

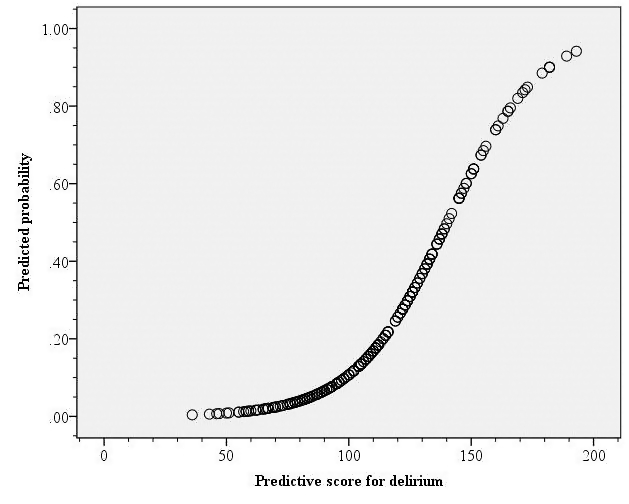

Supplement: Supplementary file 2 — Figure S4. Distribution of the predicted probability of delirious patients. (JPG 78 kb) [file 12871_2019_694_MOESM2_ESM.jpg]
